# Pulsatile Flow-Induced Fatigue-Resistant Photopolymerizable Hydrogels for the Treatment of Intracranial Aneurysms

**DOI:** 10.3389/fbioe.2020.619858

**Published:** 2021-01-20

**Authors:** Oriane Poupart, Riccardo Conti, Andreas Schmocker, Lucio Pancaldi, Christophe Moser, Katja M. Nuss, Mahmut S. Sakar, Tomas Dobrocky, Hansjörg Grützmacher, Pascal J. Mosimann, Dominique P. Pioletti

**Affiliations:** ^1^Laboratory of Biomechanical Orthopedics, École Polytechnique Fédérale de Lausanne, Lausanne, Switzerland; ^2^Department of Chemistry and Applied Biosciences, Swiss Federal Institute of Technology, Zurich, Switzerland; ^3^Laboratory of Applied Photonics Devices, École Polytechnique Fédérale de Lausanne, Lausanne, Switzerland; ^4^Institute of Diagnostic and Interventional Neuroradiology, Inselspital, Bern University Hospital, Bern, Switzerland; ^5^Institute of Mechanical Engineering, École Polytechnique Fédérale de Lausanne, Lausanne, Switzerland; ^6^Musculoskeletal Research Unit, Department of Molecular Mechanisms of Disease, Vetsuisse Faculty, University of Zurich, Zurich, Switzerland; ^7^Department of Diagnostic and Interventional Neuroradiology, Alfried Krupp Hospital, Essen, Germany

**Keywords:** pulsatile fluid flow-induced loading, intracranial aneurysms, polyethylene glycol dimethacrylate, hydrogels, fatigue, erosion

## Abstract

An alternative intracranial aneurysm embolic agent is emerging in the form of hydrogels due to their ability to be injected in liquid phase and solidify *in situ*. Hydrogels have the ability to fill an aneurysm sac more completely compared to solid implants such as those used in coil embolization. Recently, the feasibility to implement photopolymerizable poly(ethylene glycol) dimethacrylate (PEGDMA) hydrogels *in vitro* has been demonstrated for aneurysm application. Nonetheless, the physical and mechanical properties of such hydrogels require further characterization to evaluate their long-term integrity and stability to avoid implant compaction and aneurysm recurrence over time. To that end, molecular weight and polymer content of the hydrogels were tuned to match the elastic modulus and compliance of aneurysmal tissue while minimizing the swelling volume and pressure. The hydrogel precursor was injected and photopolymerized in an *in vitro* aneurysm model, designed by casting polydimethylsiloxane (PDMS) around 3D printed water-soluble sacrificial molds. The hydrogels were then exposed to a fatigue test under physiological pulsatile flow, inducing a combination of circumferential and shear stresses. The hydrogels withstood 5.5 million cycles and no significant weight loss of the implant was observed nor did the polymerized hydrogel protrude or migrate into the parent artery. Slight surface erosion defects of 2–10 μm in depth were observed after loading compared to 2 μm maximum for non-loaded hydrogels. These results show that our fine-tuned photopolymerized hydrogel is expected to withstand the physiological conditions of an *in vivo* implant study.

## Introduction

An intracranial aneurysm is an outpouching of a cerebral artery which may be present in ~4% of the population (Keedy, 2006). Aneurysm rupture is associated with a high rate of mortality and severe morbidity, thereby requires effective treatment (Medical Advisory Secretariat, 2006). Endovascular therapy, essentially coil embolization, may be performed acutely after an aneurysm rupture or electively to prevent bleeding. The treatment goal is the complete and permanent occlusion of the aneurysm while maintaining patency of the parent artery. Despite tight packing, usually <35% of the total aneurysmal volume is filled with coils (Piotin et al., 2000). This low packing density leaves room for thrombus remodeling and coil compaction, which may consequently lead to aneurysm recanalization and thus make the aneurysm prone to (re)rupture. Long-term stability is strongly correlated with the embolization volume ratio (Uchiyama et al., 2000; Tamatani et al., 2002). In addition, certain aneurysm morphologies (i.e., broad-necked aneurysms) may require advanced endovascular techniques like balloon-assisted, or stent-assisted coiling, and flow-diversion. However, compared to simple coiling, these techniques are associated with longer procedure times, higher peri-interventional risks, increased cost and require experienced operator skills.

**Figure 1:**
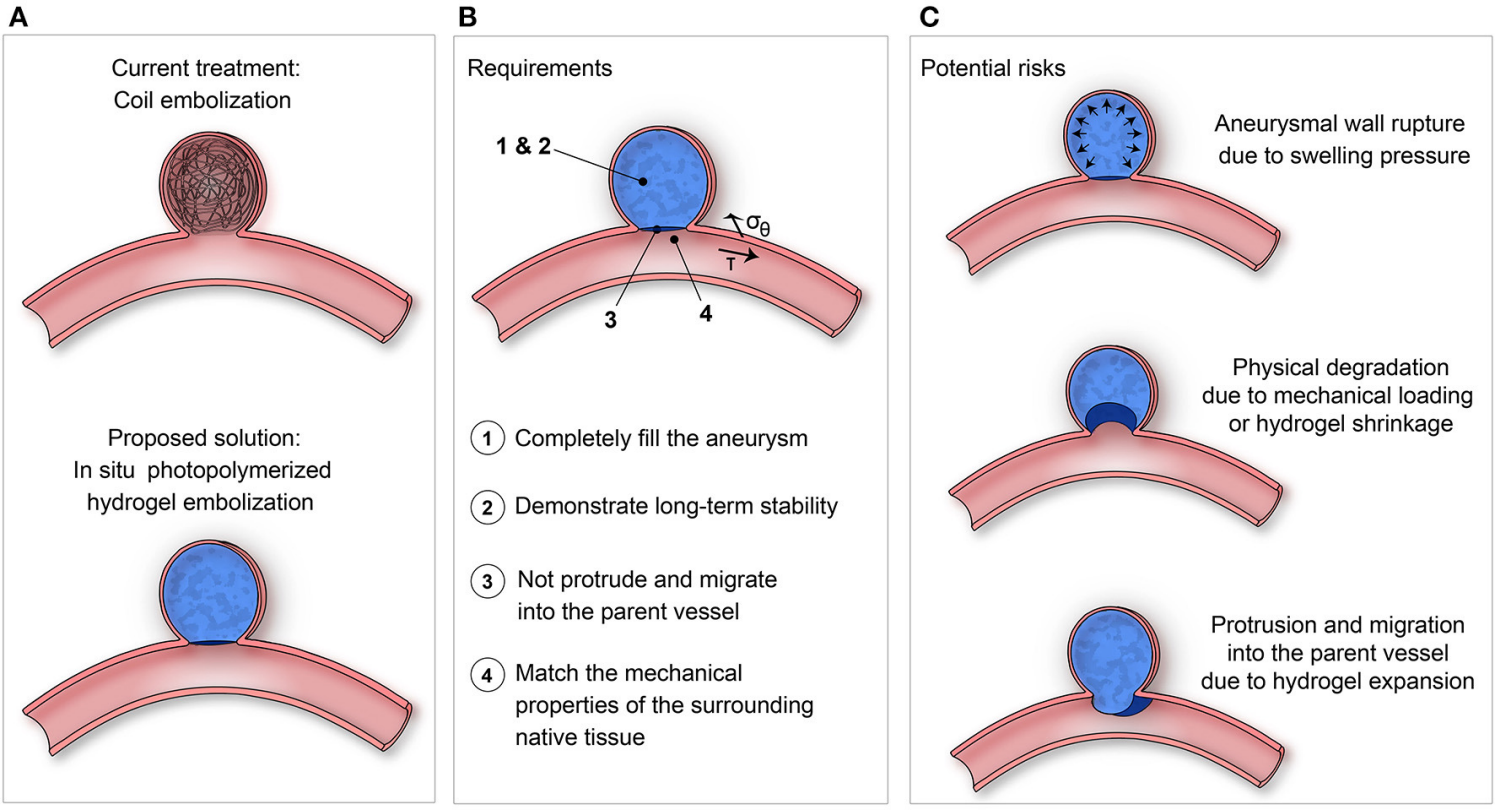
**(A)** Proposed solution of using *in situ* photopolymerized hydrogel for intracranial aneurysm embolization instead of coil embolization. **(B)** Requirements and **(C)** Potential risks to be evaluated of such a solution.

To overcome the limitations of the current endovascular techniques, the optimal embolic agent should (1) completely fill the aneurysm, (2) demonstrate long-term stability, (3) not protrude or migrate into the parent vessel, and (4) match the mechanical properties of the aneurysmal wall and surrounding tissues (Figure 1B) (Rodriguez et al., 2015).

In the past decade, various alternative embolic agents with improved packing density including hydrogel-coated coils (Taschner et al., 2018), shape memory polymer foams (Rodriguez et al., 2012; Horn et al., 2017) and *in situ* forming hydrogels have been reported (Takao et al., 2006; Barnett et al., 2009; Ishikawa et al., 2012; Bearat et al., 2013; Brennecka et al., 2013; Brady et al., 2017). Photopolymerizable hydrogels provide better spatial and temporal control over curing compared to hydrogels responsive to chemical, temperature or pH stimuli (Corrigan et al., 2019) and allow to fill any shapes *in situ*. Photopolymerizable poly(ethylene glycol) dimethacrylate (PEGDMA) hydrogels have been studied for a wide range of tissue engineering applications (Burke et al., 2019), however their use for intracranial aneurysm embolization is limited (Barnett et al., 2009; Poupart et al., 2020). Recently, the feasibility of delivery and photopolymerization of the radiopaque hydrogel using a customized 430 μm light-conducting microcatheter was demonstrated in an *in vitro* intracranial aneurysm model and the polymerized hydrogels exhibit no toxicity for the endothelial cells (Poupart et al., 2020). Further mechanical characterization and fatigue resistance tests of the photopolymerized hydrogel, however, are required to demonstrate long-term integrity and stability before proceeding to *in vivo* testing. Therefore, in this work, we investigate whether photopolymerized PEGDMA-based hydrogels may be a potential candidate as a long-term embolic agent for intracranial aneurysms.

The swelling pressure and volume ratio of the hydrogels are critical factors. Since aneurysm rupture can occur when wall stresses exceed tissue strength, hydrogel swelling and expansion must be sufficient to fill the cavity but as minimal as possible to avoid exerting excessive pressure against the aneurysm wall or protruding into the parent artery to avoid thromboembolic complications (Figure 1C). On the other hand, premature hydrogel degradation, deformation or shrinkage may favor aneurysm recurrence (Wang et al., 2019), underlying the importance for the polymer to withstand the continuous dynamic biomechanical load related to pulsatile blood flow (Wu et al., 2018). Ideally, the compliance, mechanical strength and elasticity of the hydrogel should match those of the parent artery (Tortoriello and Pedrizzetti, 2004; Morris et al., 2013). In other words, preservation of the photopolymerized hydrogel's original shape, weight and mechanical properties is crucial to ensure long-term results (Kamata et al., 2015).

In this study, molecular weight and polymer content of PEGDMA hydrogels were adjusted to fulfill the requirements of aneurysm embolic agents. Their swelling and mechanical properties were tested in static conditions and their fatigue resistance was evaluated within an *in vitro* aneurysm model dynamically stimulated by a pulsatile flow.

## Materials and Methods

### Sample Preparation

#### Materials

PEGDMA 2 and 6 kDa were synthesized from poly(ethylene glycol) (PEG, M_n_ = 2,000 and 6,000 g/mol) purchased from Sigma Aldrich (Merck, Switzerland) according to a previously reported procedure with subtle modifications (Lin-Gibson et al., 2004). The ^1^H-NMR spectrum revealed a degree of methacrylation beyond 90%. PEGDMA 20 kDa was purchased from Polysciences (Germany).

The light-sensitive and water-soluble photoinitiator, known as poly(ethylene glycol) substituted bis(acyl)phosphane oxides (PEG-BAPO), was synthesized by phospha-Michael addition, as previously reported (Wang et al., 2018).

The iodine-based contrast medium Accupaque (350 mg/ml, GE Healthcare, Chicago, IL, United States) was provided by the Bern University Hospital and the intravenous fat emulsion Intralipid 20% was purchased from Sigma Aldrich (Merck, Switzerland).

#### Hydrogel Synthesis and Preparation

PEGDMA (10, 15, or 20 wt%) with the molecular weights of 2, 6, or 20 kDa and PEG-BAPO (0.1 wt%) were dissolved in equivalent weight concentrations of phosphate buffered saline (PBS) and Accupaque. The hydrogel precursors were homogenized by Vortex and degassed using a vacuum pump. Then, the precursors were sterilized through a 0.22 μm syringe-filter (Rotilabo, Car Roth, GmbH). Finally, intralipids (5 wt%) were added to the precursors. Hydrogels were termed PEGDMAak-b, where a represents the molecular weight, k = kDa and b the concentration of PEGDMA polymer. All samples were illuminated for 10 min at a wavelength of 405 nm and an intensity of 15 mW/cm^2^.

### Material Characterization

#### Swelling Ratios

Hydrogel precursor samples were photopolymerized either in cylindrical molds before being extracted or within intracranial aneurysm models and kept inside them. The samples were immersed in PBS at 37°C and 5% CO_2_, resulting in either a free swelling or constrained swelling with the exposure to the PBS solvent only at the aneurysm neck. The swelling study was performed during 1 month and the weight and volume swelling ratios, SR_w_ and SR_vol_, at the time point t were determined using the following formulas:

$$\begin{array}{l} \text{ S}{\text{R}}_{\text{w}}\left[\text{\%}\right]=100\left({\text{w}}_{\text{t}}-{\text{w}}_{0}\right)/{\text{w}}_{0}\\ \text{S}{\text{R}}_{\text{vol}}\left[\text{\%}\right]=100\left({\text{V}}_{\text{t}}-{\text{V}}_{0}\right)/{\text{V}}_{0} \end{array}$$

where w_0_, V_0_, w_t_, and V_t_ correspond to the weight and volume of the samples after synthesis and the weight and volume of the swollen samples after an immersion of a duration t, respectively. Volumes are calculated with Archimedes' principle by immersing the samples in extra pure hexane (99+%, Fisher Chemical) for free swelling and in pure ethanol for constrained swelling to avoid the PDMS swelling in hexane.

#### Swelling Pressure

The pressure exerted by the swollen hydrogels was measured using a confined compression setup (Schmocker et al., 2016). After synthesis, 0.1 N pre-load was applied on the sample to ensure proper confinement. The chamber was filled with PBS to enable the swelling of the samples and the load was monitored while keeping the displacement constant during 48 h to reach the equilibrium swelling. The swelling pressure was defined as the maximum pressure reached during the test.

#### Mechanical Testing

Mechanical properties of the hydrogels were evaluated using an Instron E3000 linear mechanical testing machine (Norwood, MA, USA) equipped with a 250 N load cell.

Hydrogel samples were compressed to 50% applied strain at a constant speed of 0.1 mm/s. Load and displacement were recorded and the elastic modulus E was calculated by linear regression of the true stress-strain curve between the strains of 10–20%. Samples were immersed in PBS and placed in the incubator at 37°C and 5% CO_2_. After different immersion time points, the same compression test was repeated.

As-prepared hydrogels (after synthesis) were also compressed to 80% applied strain. Failure stress and strain were defined as the maximum stress and strain, respectively, before hydrogel breaking.

The compliance of the hydrogels was determined and defined as the strains applied under pressures between 10.5 and 16 kPa, corresponding to the physiological systolic blood pressure (80–120 mmHg).

### Implantation of the Hydrogel in an *in vitro* Intracranial Aneurysm Model

#### Design of *in vitro* Intracranial Aneurysm Model

**Figure 2:**
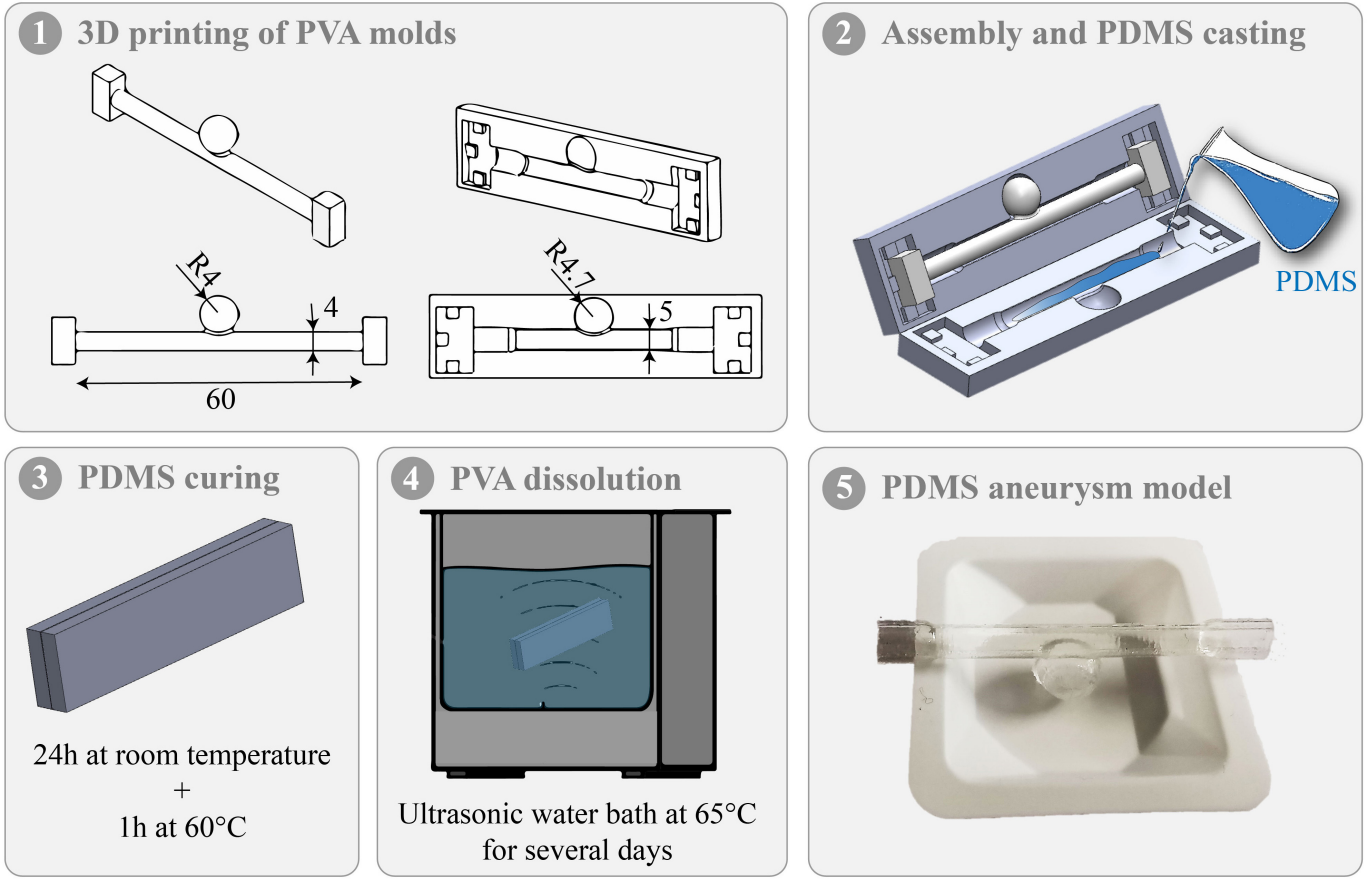
Different steps of the *in vitro* intracranial aneurysm model fabrication.

The *in vitro* intracranial aneurysm model was designed by casting polydimethylsiloxane (PDMS) around 3D printed water-soluble sacrificial molds. Specifically, sacrificial molds were first produced from polyvinyl alcohol (PVA, Digitec, Ultimaker Natural PVA filament, 2.85 mm) using a 3D printer (Ultimaker 3, Netherlands). A thin layer of water was applied on the PVA molds to smooth the surface. Secondly, PDMS (Dow Corning, Sylgard 184) with a 10:1 mixture of base to cross-linker was prepared and degassed in a vacuum chamber before being casted into the assembled PVA molds. The assembly was squeezed with a vise grip at room temperature for 24 h and then placed at 60°C for 1 h to achieve complete PDMS curing. Finally, the assembly was placed in an ultrasonic water bath at 65°C for 3 days to enable the PVA molds to dissolve. Figure 2 shows the different fabrication steps and an example of *in vitro* intracranial aneurysm model.

The elastic modulus of the models was determined by a traction assay using tensile molds in Teflon designed according to ASTM D412 (2006). In addition, the wall thickness of the aneurysm and the parent vessel was measured at 4 different locations for each model using a caliper with a measurement accuracy of 0.02 mm.

#### Implantation and Long-Term Pulsatile Fluid Flow-Induced Loading

To test the performance of the hydrogel, the hydrogel precursor was photopolymerized for 10 min in the aneurysm model using an optical glass fiber (105 μm, 0.22 NA, 1.5 m, ThorLabs, Newton, NJ, United States), proximally connected to an illumination module composed of 405 nm, 200 mW light power laser diodes (Poupart et al., 2020). The intensity at the tip of the fiber was 15 mW/cm^2^. The aneurysm model was connected to an *in vitro* flow system which was filled with a mixture of glycerol and water in a 1:1.78 ratio with 0.134% PicroSirius Red dye to mimic the light-absorbing properties of human blood. A peristaltic pump (Masterflex, ColeParmer, Vernon Hills, IL, United States) was used to generate a pulsatile flow and set to 220 ml/min to simulate human internal carotid artery flow rate (Zarrinkoob et al., 2015). The intracranial arterial pressure [91.2 ± 9.6 mmHg (Netlyukh et al., 2015)] was induced by a vertical 1.2 m liquid column. The hydrogels were dynamically loaded at 2.3 Hz for 1 month, corresponding to 5.5 million cycles in total.

#### Characterization After Loading

After the cyclic loadings, the aneurysm model was dismantled to collect the hydrogel sample. To evaluate the performance of the hydrogel, (i) the sample was weighed, (ii) the surface profile of the hydrogels examined under 3D laser scanning microscopy (VK-X200 Keyence with a 50x lens), and (iii) cylindrical samples, obtained by a 5 mm diameter punch, were compressed using the mechanical test as described above. The results were compared to the hydrogel in the as-prepared state (after synthesis) and to the hydrogel in the swollen state (after 1 month of swelling).

### Statistical Analysis

Each experiment was performed in triplicate or more and the statistical data analysis was performed with MATLAB (Mathworks, Natick, MA, United States). All data are expressed as mean ± standard deviation. One-way analysis of variance (ANOVA) was used for comparison. *P* < 0.05 was considered as a significant result (denoted as ^*^) and *p* > 0.05 non-significant (denoted ns). *P* < 0.01 was denoted as ^**^ and *P* < 0.001 as ^***^.

## Results

### Effect of PEGDMA Molecular Weight

PEGDMA hydrogels with different polymer molecular weights (2, 6, and 20 kDa) and a concentration of 10% expressed a different volume swelling and elastic modulus.

**Figure 3:**
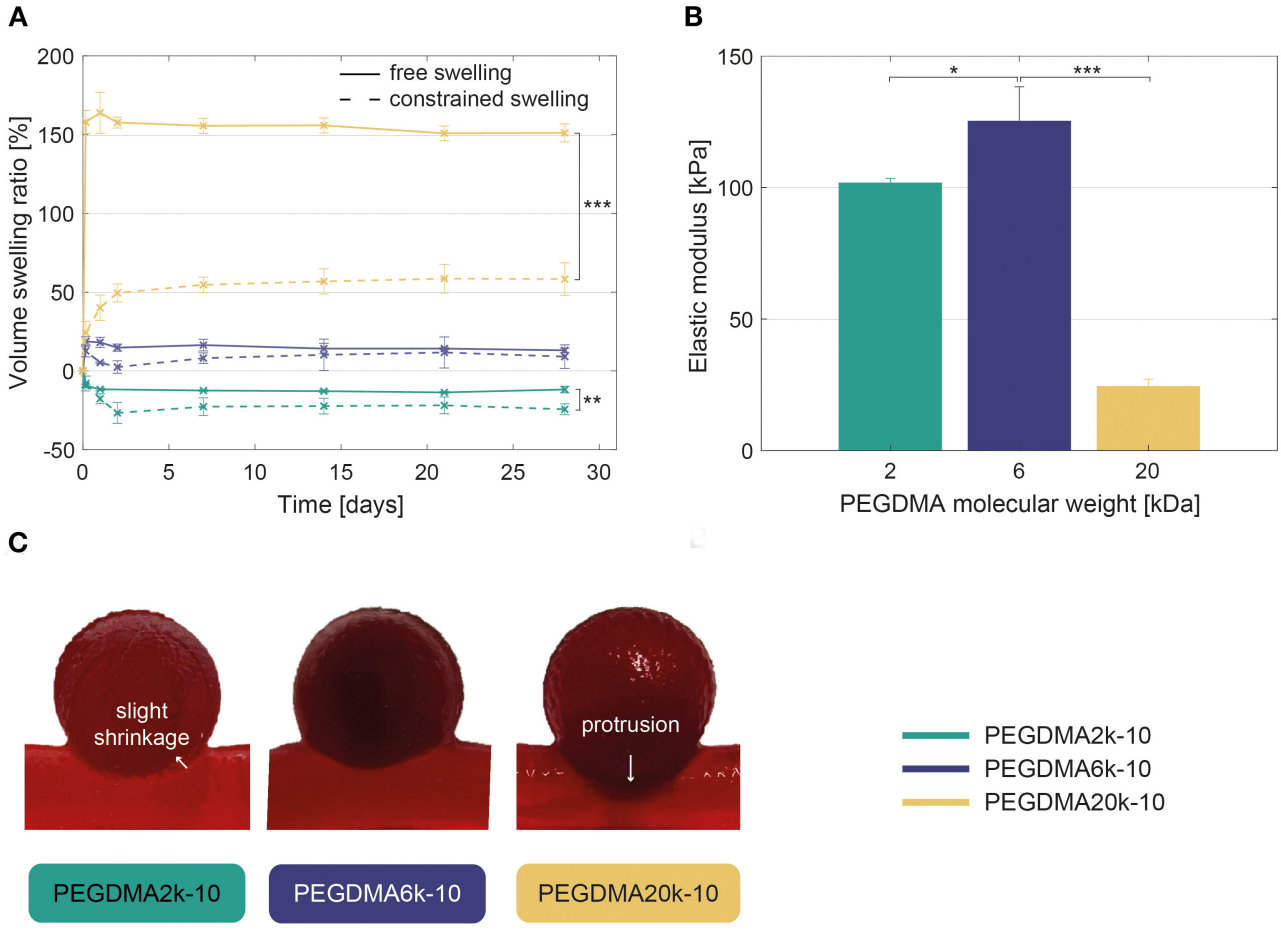
**(A)** Volume swelling ratio in free and constrained (within the aneurysm model) conditions, revealing a significant increase of the volume swelling ratio when increasing PEGDMA molecular weight. Statistically significant differences are only displayed between the free and constrained swelling conditions. **(B)** Compressive elastic modulus of the hydrogels demonstrating higher elastic modulus for PEGDMA6k-10 compared to 2–20 kDa hydrogels. **(C)** Direct visualization of the hydrogels within the intracranial aneurysm model after 1 week of fluid flow-induced loading showing slight shrinkage of the PEGDMA2k-10 hydrogel at the neck and excessive protrusion of the PEGDMA20k-10 hydrogel. *p < 0.05, **p < 0.01, ***p < 0.001.

The swelling ratio increased with the PEGDMA molecular weight and remained stable over the course of the experiment of 28 days (Figure 3A). In free conditions, PEGDMA2k-10 samples showed a slightly decreased volume (−12 ± 2%), while PEGDMA6k-10 and PEGDMA20k-10 samples manifested slightly (13 ± 1%) or extremely (151 ± 6%) increased volume swelling, respectively. The swelling ratio was significantly reduced within the constrained aneurysm model compared to free samples (e.g., 58 vs. 151% for PEGDMA20k-10, respectively), due to the limited medium absorption into the network of the hydrogel. This swelling behavior has led to a slight shrinkage of the hydrogel at the neck (PEGDMA2k-10), a moderate (PEGDMA6k-10) or excessive (PEGDMA20k-10) protrusion of the hydrogel into the parent artery. This observation was confirmed when loading the hydrogel under fluid flow, as shown in Figure 3C and Supplementary Movies 1–3.

PEGDMA6k-10 hydrogels demonstrated a higher elastic modulus compared to hydrogels with a lower or higher molecular weight (Figure 3B). PEGDMA20k-10 hydrogels withstood the maximal applied strain of 80% without failure whereas PEGDMA 2k and 6k failed under compression at a stress of 108 ± 19 kPa and 179 ± 15 kPa and an applied strain of 54 ± 4% and 65 ± 1%, respectively.

### Effect of PEGDMA Polymer Content

**Figure 4:**
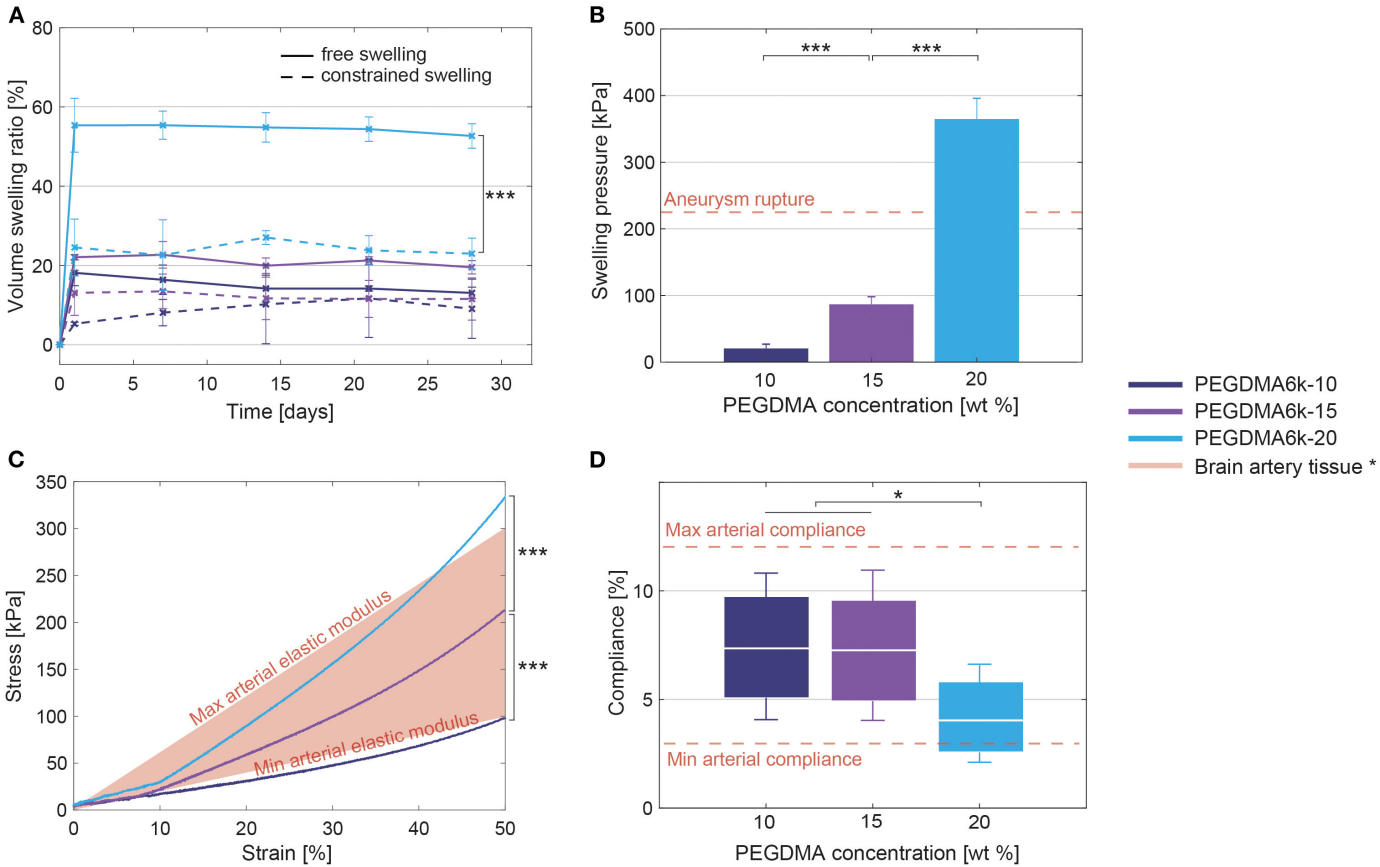
**(A)** Volume swelling ratio in free and constrained (within the aneurysm model) swelling conditions, showing a significant increase of the volume swelling ratio when increasing PEGDMA concentration. Statistically significant differences are only displayed between the free and constrained swelling conditions. **(B)** Maximal pressure induced by the swollen hydrogels increases when increasing the polymer content. **(C)** Strain-stress curve of the compression test, revealing a significant increase of the elastic modulus with the polymer content. **(D)** Compliance of the hydrogels under physiological pressures (80–120 mmHg). Box plots depicted mean (white line) and values at 80 and 120 mmHg (bottom and top of the box). PEGDMA6k-10 and PEGDMA6k-15 hydrogels have a significant higher compliance than PEGDMA6k-20 hydrogels. * parent artery tissue data from literature (Ebrahimi, 2009; Ciszek et al., 2013; van Haaften et al., 2017). *p < 0.05, ***p < 0.001.

The swelling ratio increased with the polymer content (Figure 4A), particularly in free conditions. PEGDMA6k-20 hydrogels reached a maximal swelling pressure of 365 ± 31 kPa, a three-fold increase compared to hydrogels of equal molecular weight but with a polymer content of 10–15 wt% where a pressure inferior to 100 kPa was measured (Figure 4B).

Increasing the polymer content increases the mechanical properties of the hydrogels (Figure 4C). The compressive elastic modulus was 125 ± 13, 280 ± 33, and 507 ±7 kPa for 10, 15, and 20% of PEGDMA6k, respectively. The failure stress also increased while the applied strain at rupture did not vary: PEGDMA6k at 10, 15, and 20% failed under compression at a stress of 179 ± 15 kPa, 480 ± 127 kPa, and 507 ± 125 kDa and an applied strain of 65 ± 1%, 72 ± 7%, and 64 ± 2% respectively. Compliance, under physiological pressures, was 7.37 ± 1.07, 7.25 ± 1.18, and 4.12 ± 0.60% for 10, 15 and 20% polymer content, respectively (Figure 4D).

### Fatigue-Resistance After Pulsatile Fluid Flow-Induced Loading

The mean vessel diameter and wall thickness of the designed *in vitro* models was 4.14 ± 0.069 mm and 330 ± 56 μm, respectively. The mean aneurysm diameter and wall thickness of the aneurysm was 8.45 ± 0.189 mm and 374 ± 64 μm, respectively. These values and those reported in humans are provided in Table 1 for comparison.

**Table 1:** Dimensions in our PDMS models compared to human values reported in the literature.

		**PDMS model**	**From the literature**
Parent vessel	Wall thickness [μm]	330 ± 56	460 ± 60 (Sherif et al., 2014)
	Diameter [mm]	4.14 ± 0.069	4.66 ± 0.78 (Krejza et al., 2006)
Aneurysm	Wall thickness [μm]	374 ± 64	70–530 (Blankena et al., 2016)
	Diameter [mm]	8.45 ± 0.189	3–10 (Grochowski et al., 2018)

In addition, the tensile elastic modulus was measured to be 0.662 ± 0.068 MPa, which is similar to human brain aneurysms, determined to range from 0.2 to 0.6 MPa under strains lower than 50% (Ebrahimi, 2009). Supplementary Movie 4 highlights the expansion and contraction of the *in vitro* model under pulsatile flow.

**Figure 5:**
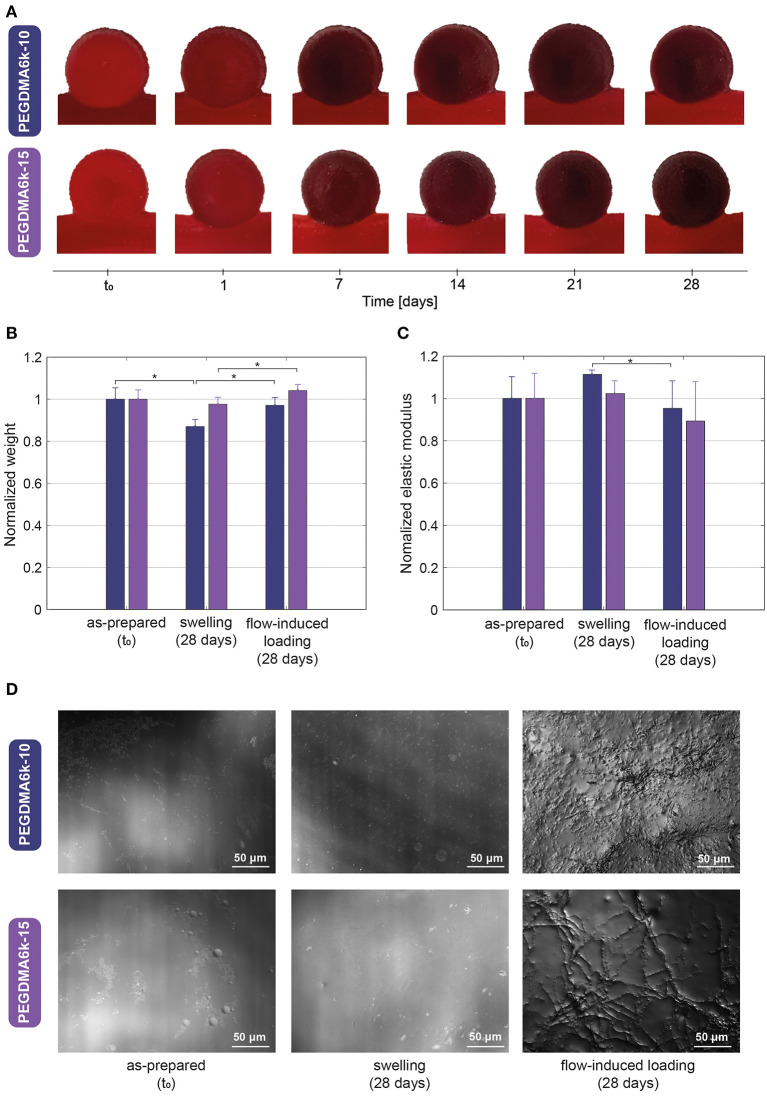
**(A)** Direct visualization of the hydrogels within the intracranial aneurysm model during 1 month of pulsatile fluid flow-induced loading showing complete occlusion without protrusion, migration, geometric distorsion nor signs of macroscopic damage. **(B)** Weight and **(C)** Mechanical property variations, normalized to the as-prepared state of the hydrogel, after 1 month of swelling and 1 month of fluid flow-induced loading. **(D)** Magnified microscopic view showing the surface profile of hydrogels in the as-prepared state (left), after 1 month of swelling (middle) and 1 month of fluid flow-induced loading (right), the latter revealing defects in the range of 2–10 μm in depth. *p < 0.05.

Figure 5A demonstrates the PEGDMA6k hydrogels with 10 and 15% content over 1 month of loading induced by blood substitute pulsatile fluid flow. The different hydrogels completely occluded the aneurysm sac without protruding or migrating excessively into the parent artery. No geometric distorsion or signs of macroscopic damage was apparent.

The weight and elastic modulus variations of the hydrogels after the flow-induced loading are presented in Figures 5B,C. After 5.5 million cycles, there was no significant weight difference between the hydrogel in the as-prepared state and the loaded hydrogel for both polymer contents. However, the weight of swollen hydrogels was significantly lower (*p* = 0.0399 and 0.0376 for 10 and 15% polymer concentration, respectively), than after 1 month of loading. Moreover, a slight decrease in the elastic modulus after cyclic loading was noticed, i.e., ~5% for PEGDMA6k-10 and 10% for PEGDMA6k-15, although this remained non-significant compared to the as-prepared hydrogels (*p* = 0.394 and 0.2111 for 10 and 15% polymer concentration, respectively).

The profile of the hydrogels surface that was in contact with the blood substitute is shown in Figure 5D. As-prepared and swollen hydrogels presented a smooth surface with minor defects of maximum 2 μm in depth, whereas loaded hydrogels demonstrated unorganized and heterogeneous roughness, with defects ranging from 2 to 10 μm in depth, as shown in Supplementary Figure 1.

## Discussion

To our knowledge, the present study is the first to assess the fatigue-resistance under physiological pulsatile flow of photopolymerizable hydrogels for intracranial aneurysm embolization. PEGDMA hydrogels, according to their polymer molecular weights (2, 6, and 20 kDa) and concentrations (10–20%), were selectively fine-tuned to fulfill the requirements of intracranial aneurysm embolic agent. The main finding of our work is that PEGDMA hydrogels of 6 kDa molecular weight at a concentration of 15% seem to be appropriate to withstand the continuous interactions with the blood flow. In fact, under pulsatile flow within an *in vitro* aneurysm model during 1 month, these hydrogels exhibited (1) a complete occlusion, (2) an integrity and stability due to the preservation of their shape, weight and mechanical properties, despite slight surface erosions, (3) a minimal swelling volume avoiding protrusion in the parent artery and a low swelling pressure limiting stresses applied on the aneurysm wall, and (4) an elastic modulus and compliance covering the range of native aneurysm tissue.

### Hydrogel Swelling and Mechanical Characterization

Molecular weight of polymers is known to affect the mechanical properties of hydrogels (Nguyen et al., 2012). The present study is consistent with and supplements PEGDMA hydrogels characterization reported in literature (Roberts et al., 2011; Khoushabi et al., 2015). An increase in polymer molecular weight decreases the cross-linking density of the hydrogel network, favoring the water diffusion through the long chains of polymer. This leads to an increase in the volume swelling ratio and a decrease in the elastic modulus. The elastic modulus of PEGDMA2k-10 hydrogels, however, was unexpectedly lower compared to PEGDMA6k-10 hydrogels. For all hydrogels, the photoinitiator PEG-BAPO concentration was fixed at 0.1 wt% for biocompatibility purposes, meaning the same amount of formed radicals. The hydrogel network is formed by a free radical polymerization of the methacrylate functional groups. PEGDMA2k-10 hydrogels have a higher number of methacrylate groups due to their shorter chain length. Consequently, the ratio between the produced radicals and the methacrylate groups is lower with PEGDMA2k-10 compared to PEGDMA6k-10. Moreover, the mobility and diffusivity of the pendant double bonds are more restricted for short chain length (Kurdikar and Peppas, 1994). This might limit further crosslinking and favor the termination reactions over chain propagation. We believe that both differences in the radicals-to-methacrylate groups ratio and kinetics of the photopolymerization can explain the lower elastic modulus for PEGDMA2k compared to PEGDMA6k. Low polymer molecular weight (2 kDa) adds brittleness to the hydrogel, as revealed by mechanical failure at stresses lower than the 100–150 kPa threshold of circumferential stresses driven by the blood pressure (Humphrey, 2008).

The swelling and mechanical properties are also influenced by the concentration of polymers (Nguyen et al., 2012). At similar molecular weight (6 kDa), increasing the polymer content increases both the elastic modulus and the volume swelling ratio, which is in line with previous reports (Lin-Gibson et al., 2004; Nguyen et al., 2012; Rennerfeldt et al., 2013; Khoushabi et al., 2018). Hydrogels with a high polymer content have a denser covalently cross-linked network in the as-prepared state, entailing a lower mesh size and more squeezed polymer chains. The swelling equilibrium is reached when the osmotic driving forces are balanced with the elastic retraction forces. When increasing the polymer concentration, the osmotic pressure also increases (Stanley and Strey, 2003) and drives solvent molecules into the hydrogel network. Due to their same molecular weight, the hydrogels reached the same swelling equilibrium and same mesh size in their swollen steady state. This implies that the hydrogels with high polymer content absorb more water and swell more to give the network more freedom to expand. PEGDMA hydrogels of 6 kDa molecular weight at a concentration of 10–20% far withstood physiologic blood pressures of 10.5–16 kPa (80–120 mmHg) and circumferential stresses of 100–150 kPa. Up to 50% strain, the load-bearing characteristic of arteries is dominated by elastin fibers, which results in the elastic modulus ranging from 200 to 600 kPa (Ebrahimi, 2009). The compressive elastic modulus of the hydrogels, ranging from 125 to 507 kPa, thereby covers the range of the native tissue, except slightly lower for a concentration of 10%. Close mechanical properties for the hydrogel and the native tissue ensure stresses caused by blood flow to be gradually transfer to the tissue and to minimize the blood flow disturbances (Rodriguez et al., 2015). The compliance match of the hydrogels and the native tissue [3–12%/100 mmHg under physiological pressures (van Haaften et al., 2017)] suggests also that the blood flow at the interface between the hydrogel and the surrounding tissue should not be disturbed (Tortoriello and Pedrizzetti, 2004; Morris et al., 2013). The swelling pressure results indicate that the PEGDMA6k-20 hydrogels, due to their increased volume swelling of 25%, apply a pressure significantly higher than the critical rupture pressure of human cerebral arteries of 224 ± 60 kPa (Ciszek et al., 2013). This would imply that PEGDMA6k-20 hydrogels would not be suitable for aneurysm embolization. It is worth mentioning that these results are in contrast to the results obtained by Hwang et al. where an expansion of 150–200% of a shape memory foam did not induce critical circumferential stresses (Hwang et al., 2012). It should also be noted that in our swelling pressure test the hydrogels were fully confined, corresponding to the worst-case scenario. The swelling pressure might therefore be lower in semi-confined conditions such as within an aneurysm. Furthermore, the hydrogels reached their swelling equilibrium after 24 h of swelling, involving that the pressurization rate is very low compared to the one used for the determination of human cerebral arteries rupture pressure (Ciszek et al., 2013). Thus, the aneurysm tissue could possibly bear this gradual pressure.

Finally, we need to acknowledge that the *in vitro* characterization was performed under fairly idealized conditions. However, this aimed to identify the hydrogels which fulfill the mechanical requirements in static conditions and exclude those inducing potential risks such as protrusion for PEGDMA20k-10 or excessive pressures for PEGDMA6k-20. The appropriate hydrogels were then exposed to a fatigue test within aneurysm models to characterize them in more realistic conditions.

### *In vitro* Intracranial Aneurysm Models

Silicone aneurysms models are commonly used to test new devices (Waqas et al., 2020), because they exhibit better transparency, resistance and navigability compared to others materials such as flexible resins like TangoPlus or VeroClear (Torres and De Luccia, 2017). The technique developed herein has been used to fabricate intracranial aneurysm models in previous studies. However, several layers of PDMS were dip-coated or painted on the sacrificial mold (Baráth et al., 2004; Liu et al., 2017), making the technique time-consuming and not reproducible. Moreover, in the previously mentioned studies, the thickness of the wall was not stated or too thick (600–800 μm). Replicas manufactured by Elastrat company (Geneva, Switzerland) are based on this technique. Although the models mimic the shape and curvature of the human vasculature, the models are very expensive (~$1,000/model), thick and stiff. The advantage of our model is the one-step casting method, the thin aneurysmal wall obtained mimicking that of native tissue, as well as similar dimensional and mechanical properties compared to native tissue not to mention a very affordable manufacturing price (~ $10/model).

In addition, the use of PVA as sacrificial mold has two main benefits. PVA is a water-soluble polymer at 65°C, thereby no toxic solvent is required for the dissolution of the sacrificial mold. Moreover, the biocompatibility of our model is not affected by the potentially infiltrated and non-dissolved PVA into the PDMS model due to the biocompatibility of PVA polymer. Comparatively, numerous studies have used acrylonitrile butadiene styrene (ABS) for the sacrificial mold which dissolves in xylene (Chueh et al., 2009) or acetone (Kaneko et al., 2016).

Although the geometry of the *in vitro* aneurysm model used in our experiments is too simple and linear to simulate complex microcatheter nagivation or deployment of devices, its mechanical properties are much closer to human tissue characteritics and thereby better suited for the long-term assessment of intrasaccular hydrogel implants. Moreover, the technique of casting PDMS around PVA sacrificial molds could be adapted to replicate more tortuous geometries to implement photopolymerized hydrogel in the same fashion as has been demonstrated in a tortuous silicone aneurysm model (Poupart et al., 2020).

### Fatigue Resistance of Hydrogels

The present study evaluated the *in vitro* fatigue resistance of the hydrogels by assessing the mechanical loss, residual mass and surface profile after mechanical loading. The hydrogels were subjected to a loading induced by pulsatile fluid flow for 1 month. This duration was determined according to the time needed for neoendothelial cells to grow over coils which generally occurs 4 weeks following implantation (Dai et al., 2016). Long-term fatigue of hydrogels is often studied under prolonged static or cyclic uniaxial loading (Bai et al., 2019). It is important to highlight that, in this work, the load-bearing ability of the hydrogels was tested under more realistic loading conditions, combining pulsatile circumferential stretch and fluid shear stresses. Indeed, similar circulating systems were used in previous *in vitro* studies to reproduce realistic blood flow and therefore closely simulate circumferential and wall shear stresses (Mokin et al., 2019; Cancelliere et al., 2020; Yamaki et al., 2020).

The results after 5.5 million cycles of pulsatile flow loading pointed that the hydrogels have a great integrity and stability owing to the preservation of their shape, weight and elastic modulus. However, mechanical loading has induced roughness and erosion of the hydrogel surface that was in contact with the fluid. The weight of the loaded hydrogels was not significantly different from the as-prepared hydrogels but higher than the 1-month swollen hydrogels. We hypothesize that it might be due to an alternating of swelling and erosion cycles. In fact, when the hydrogel has reached its swelling equilibrium weight, the hydrogel surface erodes, which decreases the hydrogel weight and enables to start again the swelling process (Makino et al., 2001). Surface erosion, forming roughness defects and micro-cracks of several microns, induces a small increase in porosity (Gade et al., 2017). This change of the surface microstructure enhances the permeability and water absorption of the hydrogels (Omidian et al., 2005; Zhao et al., 2016), which might explain the increase of weight swelling ratio after loading and hence after erosion. A further analysis of the erosion phenomena could confirm these results.

Erosion of a material is generally characterized by the residual mass and mechanical properties loss (Dalmoro et al., 2016; Gade et al., 2017) but the surface profile has not been studied. Our findings suggest that mass and mechanical loss are not sufficient to conclude regarding the erosion phenomena. Thus, an important implication of our findings is that the characterization of the surface might be of primary interest for the fatigue resistance evaluation.

A question associated with the erosion is its impact on the long-term efficiency of hydrogels as embolic agents. Even so roughness defects of loaded hydrogels are relatively low (<10 μm), the risk of hydrogel fragments moving downstream and thus the risk of stroke should not be underestimated. Another concern is that the erosion might be faster in blood. Indeed, one limitation is that the fatigue study we performed was in a fluid composed of PBS and glycerin without degradation enzymes. Nevertheless, the erosion process could also be advantageous to increase the hydrogel pores size and thus cell penetration (Bar et al., 2018), which could foster the endothelialization of the hydrogel surface along the aneurysm neck.

A limitation of our work is that the fatigue resistance was studied only for a short duration (1 month at 2.3 Hz corresponding to an implantation of 2 months *in vivo*) compared to years of implantation of an embolic agent in an aneurysm. However, an endothelial layer generally forms at the implant surface within 1 month of implantation, acting as a physical barrier to prevent direct contact between the blood and the implant (Dai et al., 2016). In case of cell adhesive properties, the hydrogels might hence be protected at its surface by the endothelial layer. Nonetheless, these properties should be further studied to determine whether the exclusion of the aneurysm from the parent artery by endothelialization will reduce the risks caused by fatigue loading.

Furthermore, we need to acknowledge that although the hydrogels remained within the aneurysm model, the adhesion of the hydrogel to the endothelial tissue cannot be elucidated in the context of the present study and needs to be further investigated in order to avoid migration of the hydrogel into the artery. For this, an *in vivo* study in an elastase-aneurysm model in rabbits will examine the tissue adhesion as well as clinical safety and long-term efficiency of the photopolymerizable hydrogels as embolic agent.

## Conclusion

Photopolymerizable PEGDMA hydrogels of 6 kDa molecular weight at a concentration of 15% demonstrated mechanical properties and compliance consistent with the native aneurysm tissue while presenting a minimal swelling volume and pressure. The hydrogels were able to withstand 5.5 million cycle of pulsatile flow loading in an *in vitro* aneurysm model without a significant change in shape, mechanical properties or weight loss, despite slight surface erosion. This might enable neoendothelial cells to proliferate and lead to permanent sealing at the neck. *In vivo* studies are required to confirm these results and evaluate the long-term integrity and stability of such hydrogels, as well as their tissue adhesion properties in true physiological conditions.

## Data Availability Statement

The raw data supporting the conclusions of this article will be made available by the authors, without undue reservation.

## Author Contributions

OP developed the hydrogels, performed the swelling and mechanical testing in static and dynamic conditions, characterized the hydrogel properties, analyzed the results, wrote the manuscript, and prepared the figures. OP and LP designed the *in vitro* aneurysm models. RC synthesized the chemical components of the hydrogels. PM and DP supervised the project and share equal senior contributions. AS, CM, PM, and DP reviewed the paper. All authors commented on the paper.

## Conflict of Interest

AS was a shareholder at Lumendo SA to which the intellectual property was licensed. Lumendo SA was not involved in the study design, collection, analysis, interpretation of data, the writing of this article, or the decision to submit it for publication. The remaining authors declare that the research was conducted in the absence of any commercial or financial relationships that could be construed as a potential conflict of interest.

## Supplementary Material

Supplementary Movie 1 PEGDMA2k-10 hydrogel after 1 week of fluid flow-induced loading.

Supplementary Movie 2 PEGDMA6k-10 hydrogel after 1 week of fluid flow-induced loading.

Supplementary Movie 3 PEGDMA20k-10 hydrogel after 1 week of fluid flow-induced loading.

Supplementary Movie 4 Expansion and contraction of the *in vitro* intracranial aneurysm model without hydrogel.
