# Supplementary figures and images for: Pulsatile Flow-Induced Fatigue-Resistant Photopolymerizable Hydrogels for the Treatment of Intracranial Aneurysms
Source: Front Bioeng Biotechnol. 2021 Jan 20;8:619858. doi: 10.3389/fbioe.2020.619858 (PMC7855579; doi:10.3389/fbioe.2020.619858)

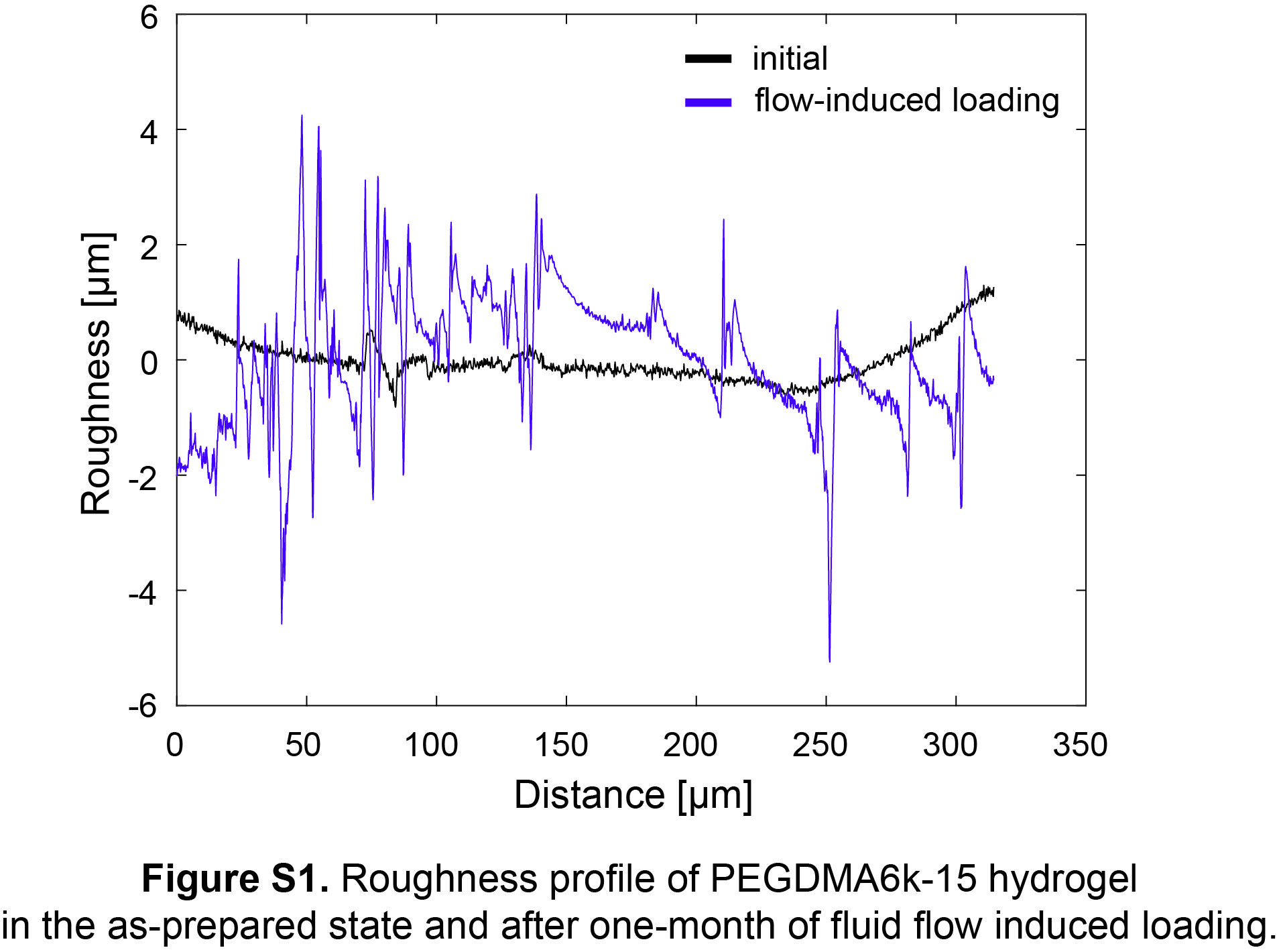

Supplement: Supplementary file 1 [file Image_1.JPEG]
